# Identifying Application Areas for Machine Learning in the Retail Sector: A Literature Review and Interview Study

**DOI:** 10.1007/s42979-023-01888-w

**Published:** 2023-06-07

**Authors:** Clemens Brackmann, Marek Hütsch, Tobias Wulfert

**Affiliations:** Universitätsstraße 2, 45141 Essen, Germany

**Keywords:** Retail, Electronic commerce, Machine learning, Application area, Literature review

## Abstract

Machine learning (ML) has the potential to take on a variety of routine and non-routine tasks in brick-and-mortar retail and e-commerce. Many tasks previously executed manually are amenable to computerization using ML. Although procedure models for the introduction of ML across industries exist, the tasks for which ML can be implemented in retail need to be determined. To identify these application areas, we followed a dual approach. First, we conducted a structured literature review of 225 research papers to identify possible ML application areas in retail, as well as develop the structure of a well-established information systems architecture. Second, we triangulated these preliminary application areas with the analysis of eight expert interviews. In total, we identified 21 application areas for ML in online and offline retail; these application areas mainly address decision-oriented and economic-operative tasks. We organized the application areas in a framework for practitioners and researchers to determine appropriate ML use in retail. As our interviewees provided information at the process level, we also explored the application of ML in two exemplary retail processes. Our analysis further reveals that, while ML applications in offline retail focus on the retail articles, in e-commerce the customer is central to the application areas of ML.

## Introduction

It is estimated that by 2025 more than 150,000 mobile robots will be deployed in retail to execute routine work such as refilling shelves and preparing package dispatch, making use of machine learning (ML) [1, 50]. However, ML cannot only be implemented to overtake cognitive routine tasks involving rule-based tasks [25], but can also spread into domains previously defined as non-routine tasks (e.g., handwriting recognition or car driving) [83]. Furthermore, ML has different applications such as X-ray image analysis [70], microorganism image analysis [98], histopathology image analysis [13] and cancer image analysis [12]. A study by Frey and Osborne [23] identified that many tasks in online and offline retail are amenable to computerization. Although retail in both offline and online environments involves a variety of tasks requiring different capabilities, a study by McKinsey revealed a high potential for automatizing in general and ML in retail [55], which is why we focused on application areas for this. The focus in retail is on primary and value-adding tasks that are described by Schütte as “economic-operative tasks” [55, 77].

Brick-and-mortar retailers have faced increasing competition in recent years, especially from online competitors. This process has been intensified in particular by the COVID-19 pandemic [63]. While the use of ML has become strongly established in e-commerce, the pressure on brick-and-mortar retailers is also increasing in this area [27]. While brick-and mortar retailers manage products, logistics, etc. at store level, online retailers focus on optimizations at the customer level [77]. E-commerce involves much data regarding customers and unstructured data regarding products (product reviews, products created by users on a marketplace). The situation in brick-and-mortar retail is different [17, 33, 65]. The majority of data is present in a structured manner and data about articles are often self-managed by product manufacturers. In addition, there is only a limited amount of data about the customers, potentially limiting the introduction of ML to only a few application areas. ML usually refers to either classification or regression, whereas classification is not relevant for our approach, although it can be used in retail, since we focus on revenue which can only be predicted with regression models.

The ML algorithm selection problem—also known as the combined algorithm selection and hyperparameter optimization (CASH) problem—poses a challenge to all ML practitioners [81]. This not only involves the selection of an algorithm that fits the data set and the economic problem of the retailer, but also the model selection and hyperparameter optimization [8]. Due to the abundance of possible combinations of these factors and their influence on the quality of the result, it is essential to choose fitting factors (i.e., algorithm, model, and hyperparameter) that fit the underlying data as best as possible [81]. Recently, however, various automated ML tools have been created for making these decisions, which greatly facilitate the real-world application of ML methods [22]. Thus, even practitioners who are non-experts in the various ML disciplines can achieve good results using the automated ML tools and available procedure models for introducing ML. Although these tools exist, it is still necessary to have experts who determine which tasks can be supported or even automated by ML. Experts also need to be able to create increasingly complex ML models themselves [33]. ML experts can be supported by industry-specific frameworks indicating possible ML applications. Such frameworks can resemble an entry point for ML implementation in online and offline retail. However, reviews in the existing literature on the application of ML in retail are usually limited to one application area and focus only on online or offline retail [10]. Against this backdrop, we propose the following research question:

*What tasks in (online and offline) retail can be supported by machine learning?*

To address this research question, we provide an overview of multiple application areas for ML in retail using a dual approach. We conducted an application-centered literature review for extracting possible ML applications for retail [88]. We triangulated these preliminary application areas with expert interviews on the usage of ML in retail processes [60]. We made use of an agreed-upon reference model in retail to structure the identified ML application areas, along with retail-specific tasks [77]. This paper contributes to the development of a customized strategy for ML in retail. Hence, we propose decision-making support for retailers on ML usage in specific application areas.

This research paper is an extended version of a paper presented at the 24th International Conference on Enterprise Information Systems [34]. For this special issue of SN Computer Science we modified the introduction, conducted an additional interview study, triangulated the literature-based application areas with state-of-the-art insights from retail practice, demonstrate our results with selected retail processes, improved the discussion section, and streamlined the concepts used throughout the text.

The remainder of this research paper is structured as follows: in section 2, we elaborate on fundamentals about retail information systems in general and ML in particular. Next, we discuss our structured literature review and expert interview approach. We present the identified application areas of ML in retail in section 4, and then we demonstrate the application of ML in retail by means of two process examples in section 5. We discuss current and future applications of ML in online and offline retail in section 6. The research paper concludes with a brief conclusion and an outlook for future research.

## Theoretical Background

In this section, we uncover related literature for retail information systems and machine learning.

### Retail Information Systems

Since the advent of the Internet, existing retailers have either extended their traditional in-store business by an additional online channel or new companies that have started up have implemented an e-commerce business model, selling articles online without operating any brick-and-mortar stores [73]. Regardless of the institutional-economic discussion on retail companies, the tasks intended for trading functions are economically necessary [47]. There are three basic functions bridging the gap between manufacturer and customers in the streams of real goods (goods, services, returns), nominal goods (money, credits), and information across space, time, quantity, and quality [5]. Facilitated by the ongoing digitalization of the retail sector, the three basic functions increasingly cope with digital product and price information, payment, logistics, and distribution processes [48].

**Fig. 1:**
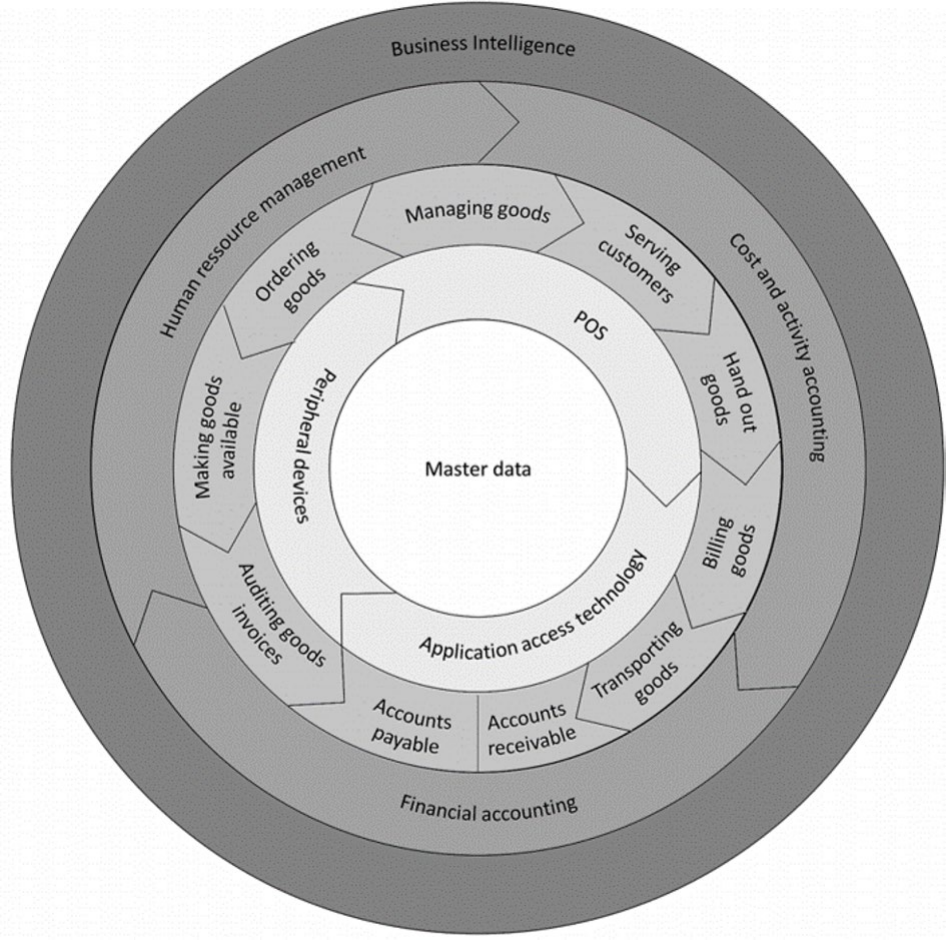
Retail Information System Architecture [34, 76, 77]

In e-commerce, trading transactions are carried out digitally to some degree [47]. The degree to which transaction must be digitalized varies in the literature on a continuum between a completely digital transaction and digital functions constituting only a small part of the procurement process [47]. Information systems in retail support the execution of the three trading functions and related tasks. They support the operational-dispositive, the business administration-administrative, and the controlling, as well as corporate planning tasks [6]. They extend the components of merchandise management systems (merchandise planning, logistics, and settlement processes) by using business intelligence and necessary corporate-administrative tasks in an integrative manner to conduct the business processes of a retail company [6, 77]. The necessary tasks of a retailer and supporting information systems can be depicted in process models and reference models for the retail industry. Reference models comprise a high-level sketch of the system. They can depict the application architecture of a specific company or only part of its application architecture [29]. The current body of literature includes a number of reference models describing retail information systems [3, 6, 76]. In this article, we focus on the shell model, as proposed by Schutte, as it covers the whole value chain, from manufacturer, wholesaler, and retailer to the end consumer, focusing on tasks at the business level, and is designed for both online and offline environments. The shell model is a task-oriented reference model for retail [76]. We use this task-oriented perspective to determine the tasks for which ML is already applied, according to the scientific literature. It follows the principle of the separation of tasks and actors, introduced by Ferstl and Sinz [21]. The shell model copes with the identified shortcomings of the H-model, such as the artificial separation between merchandise management and decision support systems. It consists of four separate but intertwined architectures for each of the aforementioned actors along a value chain. The retail architecture consists of five shells for the main retail tasks (master data, technical tasks, economic-operative tasks, administrative tasks, and decision-oriented tasks) of each actor, and the retailer in particular [77]. Each shell consists of a series of tasks that form the components of the architecture. The tasks relevant to structuring the application areas are described in the analysis section (Sect. 4). The shell model is depicted in Fig. 1.

The master data on the article, supplier, customer, condition, site, and warehouse form the basis for technical tasks, peripheral control, and user access technology. The economic-operative tasks consist of merchandise management, in which the purchasing and sales sides are brought together by uniform marketing. The product range must be defined, the suppliers must be selected, the purchase price is negotiated, and the sales prices and sales performance are determined [92]. Based on the tasks of merchandise management, procurement and distribution processes are controlled with the functions ”plan,” ”provide,” and ”check invoice.” The distribution of goods or the sales process culminates in serving the customer. The cashing and invoicing processes follow serving and lead to the accounts receivable accounting. The goods are then transported to the customer. Additionally, inventory management as part of managing goods refers to the management and optimization of goods and storage, respective the inventory. The administrative tasks consist of the three basic processes of financial accounting, cost and activity accounting, and human resource management. The decision-oriented tasks (i.e., business intelligence) provide aggregated information to the management of a retailer [77].

### Machine Learning

Historically, computerization has largely been confined to manual and cognitive routine tasks involving explicit rule-based activities [25]. Following recent technological advances, ML is now spreading to domains commonly defined as non-routine [83]. As these non-routine tasks are commonly executed by human employees, this paper reviews research articles to investigate which of these non-routine tasks in offline and online retailing are supported by ML in terms of research and are thus potential candidates for supporting humans with ML in the future.

**Fig. 2:**
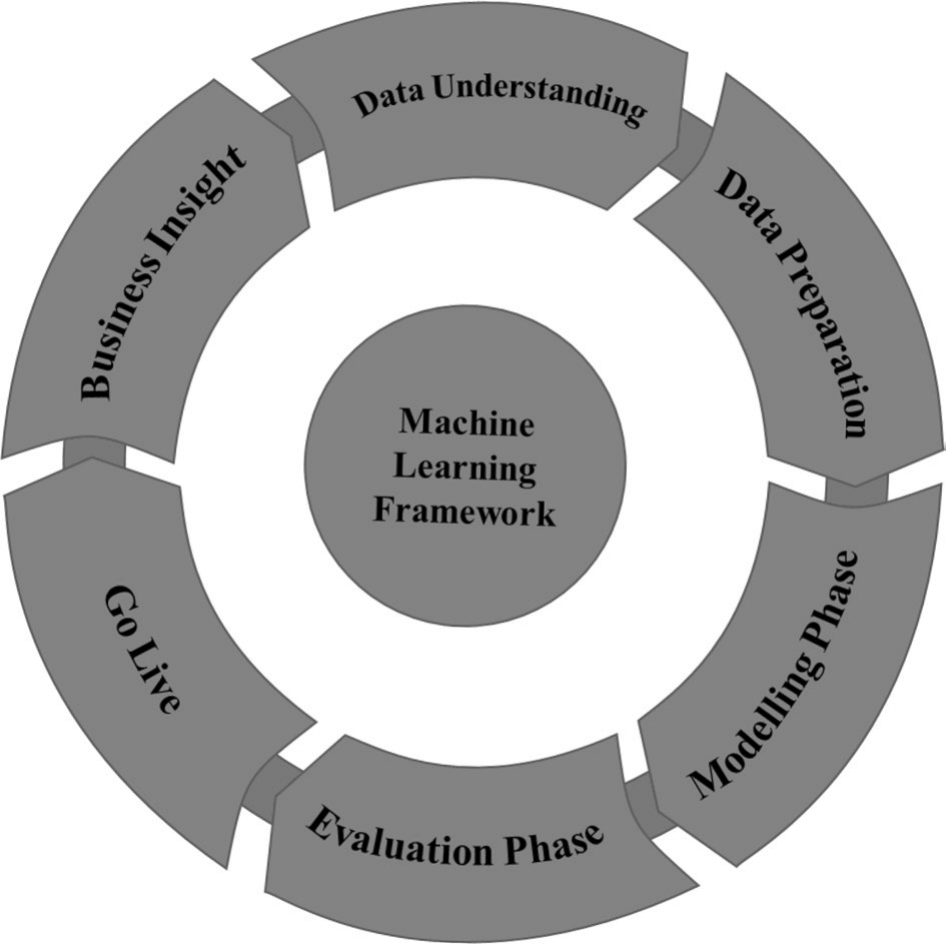
Typical Machine Learning Framework inspired by Nguyen et al. [62]

The definition of ML we use describes ML as follows: “a computer program is said to learn from experience E with respect to some class of tasks T and performance measure P, if its performance at tasks in T, as measured by P, improves with experience E” [61]. To fulfill this definition, an ML application needs an optimization algorithm, an error function, a model, and a data set. A common way of using ML is illustrated by the framework shown in Fig. 2. This framework consists of six phases, where the first two phases, *Business Insight* and *Data Understanding* focus on the understanding of the context and data where the ML application will be used. The *Data Preparation* phase focuses on the implementation of algorithms for data augmentation and transformation, as well as the preparation for their use in the following phase, the *Modeling Phase*, where the actual implementation and selection of the ML model and algorithm take place. Next, the Evaluation Phase focuses on testing and refining the previously created ML models, and finally, the *Go Live* phase focuses on the introduction of the ML application in the intended context [62]. In the specific case of retailing, it is the introduction in retail-related business processes with the intention of improving process efficiency and effectiveness.

As we use some ML vocabulary in the remaining paper, we clarify these terms below. A “forecast” is defined by historical time-series data that are used to make assumptions about possible events in the future. A ‘classification algorithm” makes statements about a data set and assigns these data to a given set of classes/labels. There are many other ML tasks, which are mostly incorporated with a combination of forecasts and classification algorithms, which we mostly refer to as "*analysis*" in this paper. In a similar work, Weber and Schütte [93] inductively determined that artificial intelligence use cases for brick-and-mortar retail from real-world examples. Although the broader term of artificial intelligence (AI) is used there, application areas of ML are also included. Just as in this paper, Weber and Schütte [93] used the shell model as a starting point for the literature review (Fig. 1). Both papers complement each other by identifying ML applications from practical contributions [92] and from analyzing scientific contributions. The different approach of this paper makes sense, as the underlying research question is another one. In this paper, the potential application areas of ML in retail are reviewed by analyzing research papers as a framework for practitioners and researchers. Another related work by Bousqaoui et al. [10] focused on the use of ML in the domain of supply chain management.

## Scientific Approach

To identify relevant application areas of ML in retail (online and offline), we followed a dual approach. We identified possible application areas of ML in retail from the scientific literature by performing a systematic literature review [88]. In addition, we augmented these theory-driven application areas with eight interviews with domain experts [60].

### Systematic Literature Review

To determine the theory-driven application areas of ML in retail, we followed the structured literature review approach proposed by vom Brocke et al. [88]. Following Cooper [15], our literature review can be characterized as follows: We focused on research outcomes (use cases and ML algorithms for retail) and applications of ML in retail. We aimed to integrate the body of literature and generalize application areas from the available research. We objectively represent articles from our exhaustive literature review with citations of selected papers for each application area. In this paper, we collect results from previous works and integrate them into an overarching framework. Our research represents scholars specialized in the use of ML in retail and e-commerce and practitioners implementing ML algorithms.

**Fig. 3:**
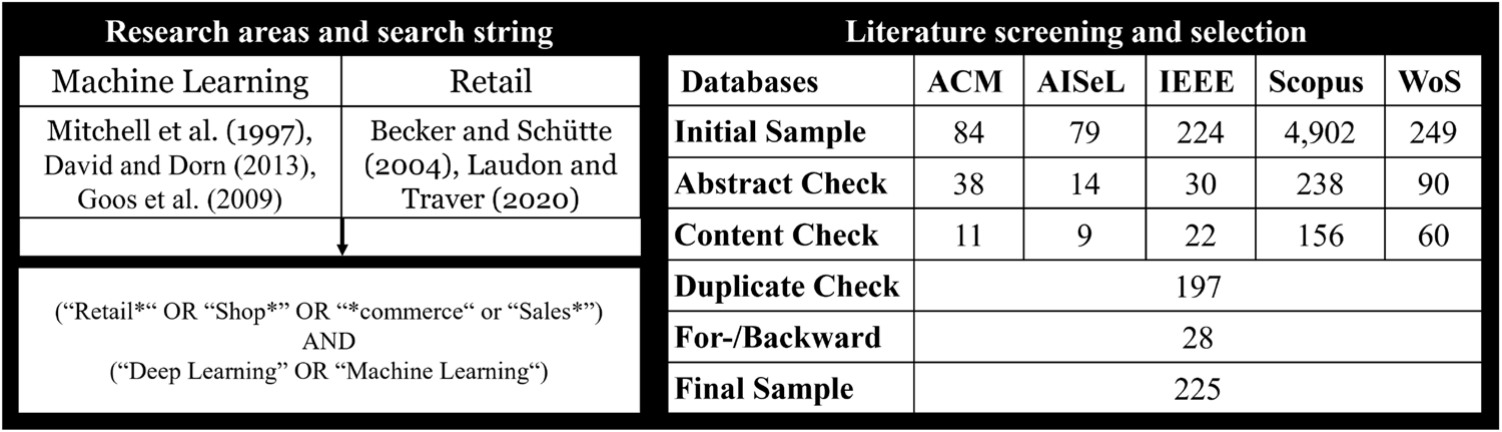
Structured literature review design [34]

We identified keywords from the related literature, and an initial literature screening resulted in a generic search query that consisted of a retail and an ML part (see Fig. 3). As we derive existing application areas of ML in retail from relevant journals and conference papers and propose avenues for further ML application in this domain, we chose a broad literature scope. This broad scope was underpinned by the search engines SCOPUS, Web of Science (WoS), IEEE Xplore (IEEE), the AIS electronic library (AISeL), and ACM. We queried their underlying databases, which consist of research from, among others, the economics and computer science literature. An initial query for titles and keywords on 2021-04-15 resulted in an initial sample of 5,538 papers. To ensure an appropriate level of quality, we focused on the scientific literature and added additional quality criteria to the search [71]. We excluded non-English and non-German language articles; panels and commentaries; purely technical articles (e.g., articles that focus exclusively on technological aspects without applying them in an e-commerce context); and articles with a purely e-commerce focus (e.g., articles that focus exclusively on e-commerce or sub-types without the inclusion of conceptual models). In contrast to previous research, such as Weber and Schutte [92], we intentionally excluded white papers and practitioner reports. Based on the title and abstract, considering the quality criteria, we reduced our sample to 410 papers. Further applying the approach of Bandara et al. [4] to the content of the papers, we identified 261 relevant publications, leaving 197 after the exclusion of duplicates. We used a one-time forward and backward search to identify research presented as an alternative scenario, resulting in 28 additional papers and resulting in a final set of 225 papers (see Fig. 3).

**Fig. 4:**
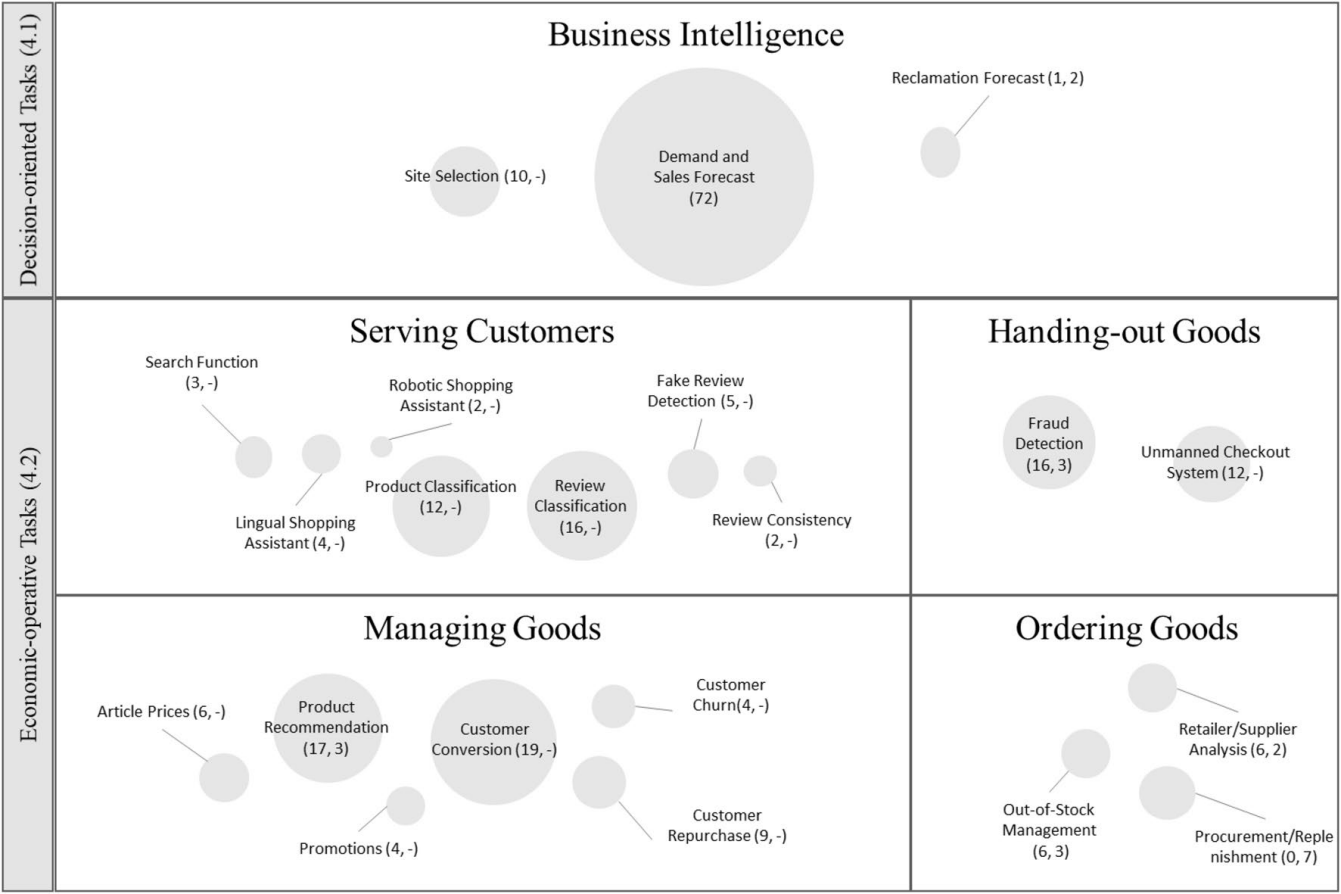
Framework for Machine Learning Application in Retail [34]. The tuples show the results of the literature review (left) and the interviews (right)

The articles in the final set were independently analyzed by full-text screening, and the relevant text passages, including possible application areas, were coded to refine the set of application areas. These application areas were synthesized to ensure the accuracy of our approach. The shell model, with its central master data objects (first shell), technical tasks (second shell), operational tasks (third shell), administrative tasks (fourth shell), and decision-oriented tasks (fifth shell) [76], served as the basis for the coding, which was inductively adapted as needed. The initial set of possible application areas was refined, using our final set of papers and two independent researchers in three coding rounds. After each round, the researchers met via online communication media to discuss the application area refinements. The framework in Fig. 4 depicts our preliminary results, including 20 theory-driven application areas for ML in retail.

### Expert Interviews

The preliminary application areas identified by the literature review were augmented by a series of interviews with domain experts [75]. During the interviews, we addressed the application of ML in retail-specific processes in a semi-structured manner. In total, we conducted eight interviews and discussed a total of seven processes. To create as broad and diverse a knowledge base as possible, we generalized information from a diverse set of interviewees [35]. The experts work at consulting agencies, with online and offline retailers as clients, and in close cooperation with software providers, whose software they implement. The interviewees have an average experience of 18 years as consultants in retail in a variety of process areas (e.g., finance, sales and distribution, forecast, and replenishment). The interviews were conducted online between 2022-06-08 and 2022-06-30 on regular workdays between 12:00 and 17:00, with an average interview duration of 59 min. Descriptive information about the interviews are presented in Table 1.

**Table 1:** Overview of interviewees

#	Interviewee’s role	Processes	Process area	Domain experience	Date	Time
1	Senior consultant	Audit	Finance	$\sim$9 years	22-06-08	12:02–12:59
2	Managing partner	Returns	Sales & distribution	$\sim$23 years	22-06-08	13:15–14:19
3	Senior consultant	Procurement	Forecast & replenishment	$\sim$6 years	22-06-13	12:32–13:27
4	Senior consultant	Inventory planning	Sales & distribution	$\sim$20 years	22-06-16	16:01–16:57
5	Senior consultant	Promotions	Sales & distribution	$\sim$27 years	22-06-22	14:36–15:36
6	Solution architect	Distribution	Sales & distribution	$\sim$29 years	22-06-23	12:01–12:58
7	Consultant	Contingency	Sales & Distribution	$\sim$16 years	22-06-27	13:34–14:39
8	Consultant	Distribution	Sales & distribution	$\sim$12 years	22-06-30	15:29–16:25

Our semi-structured interviews were based on a previously known guide to ensure that all conversations covered a similar range of questions regarding the investigated processes while allowing for the greatest possible flexibility in exploring explicit knowledge [58, 60]. In addition to demographic data, we also asked about the role of the interviewees within the companies, the retail sector they work in and their ML knowledge. Then we focused on the processes in which ML can be used. The rest of the interview was conducted through exploratory questions. We defined four leading questions for the interviews: "In which scenario can AI be applied?", "What processes are part of that scenario?", "What are the relevant input data for this scenario?", and "What is the value added through the usage of ML in this scenario?“. The semi-structured nature of the interviews allowed us to check back certain scenarios and allowed the interviewees to elaborate on selected processes in detail. The processes investigated depended on the process area in focus for each interviewee. The interview guide was created based on our research question, the shell model as reference model, and the preliminary set of application areas identified in the literature review. The interviews were conducted via electronic communication media, such as Zoom and Microsoft Teams.

For the knowledge extraction, the interviews were anonymized and transcribed in order to perform a qualitative content analysis, which aimed at systematically analyzing the object of communication, regardless of its form [75]. Sentences were chosen as coding units, while a paragraph of an interview were regarded as a context unit [56]. Compound sentences and sentences assignable to more than one category were coded multiple times [56, 94]. The first is the smallest possible segment which can be coded, while the latter is the largest codable segment. The interview analysis was supported by MAXQDA [41]. We used our theoretically derived application areas as the initial categorization system and iteratively adjusted this scheme with our eight expert interviewees. In total, we identified 21 application areas for ML in (online and offline) retail, as shown in Fig. 4.

As we not only discussed the use of ML in retail at application area level but also at process level, we demonstrate the use of ML in two selected processes discussed with the domain experts. We followed the approach presented by Weber and Schütte [91]. Consequently, we depict the restocking process and the returns process in the Business Process Model and Notation (BPMN) [67] and indicate activities that can be supported or even replaced by the application of ML. We refer to Becker and Schütte [6] for the reference process activities and events for both processes.

## Application Areas of Machine Learning in Retail

This section presents the application areas for ML, which resulted from our structured literature review and the analysis of the interviews with the domain experts. The assignments between business tasks of the retail shell model and ML application areas are illustrated in Fig. 4. The number of identified scientific papers is presented between brackets in Fig. 4 for each application area on the left, and the number of processes discussed in the interviews are presented on the right. Each application area is assigned to a task of the shell model. Each shell of the shell model for retail consists of a series of tasks that form the components of the architecture. The master data article, supplier, customer, condition, site, and warehouse are the basis for technical tasks, peripheral control, and user access technology. We present each application area in general, and the results of the literature review are described specifically by outlining a sample of the literature review results. We also elaborate on the processes discussed during the expert interviews and the specific application of ML.

### Decision-Oriented Tasks

The decision-oriented tasks (i.e., business intelligence) of the shell model provide aggregated information to the management of a retailer [78]. One very important business intelligence ML application area is the sales forecast, which is represented by 72 papers. The sales forecast provides management with information on future customer demands, and therefore management is able to plan operating activities accordingly.

*Sales forecasting* per article is one of the ML areas in retail that receives the most attention, because it is the basis for numerous advanced algorithms, such as price optimization [11] and promotion optimization [30]. Forecasting techniques are divided into four main groups: qualitative methods which are based on human judgment, time-series methods that use historical data, causal methods that use rule-based forecasts, and simulation methods that simulate the behavior of the customer [14]. If the focus is on-demand forecasting with ML methods, time-series methods are mostly used. The forecast for individual days has the difficulty that rare events are decisive in the prognosis, which are often hardly considered as outliers at weekly or monthly levels [31]. The demand forecast can be extended by features; for example, Verstraete et al. [86] created short-term and long-term forecast models that take weather data into account. Loureiro et al. [52] compared demand forecasts in the context of the fashion industry, where short product life cycles are evident.

The inverse of a sales forecast is the *forecast of returned goods*. For example, when it comes to product recalls, management must factor a high volume of returned products into operational activity planning. In addition, the returns process deals with goods that have been complained about. This can happen in case of wrong deliveries, faulty goods, or even defective goods [6]. Kumar et al. [44] used multiple ML algorithms for the *reclamation forecast*. This forecast is integrated into the supply chain design and planning problems to optimize supply chain tasks. The expert interviews confirmed that ML can be used to predict returns per customer. This involves using historical returns data to evaluate customer profiles and determine the probability of whether a return may occur. As a result, customers are assigned a score accordingly and prioritized in future ordering processes (Interviewee 2). Furthermore, using ML, so-called returns clustering by the supplier can be performed. In this process, supplier returns are analyzed and evaluated accordingly within the procurement process to make a statement about the delivery reliability of a supplier (Interviewee 3).

A store is an organizational unit used to document the goods in stock for inventory management and to map the associated business processes, such as goods receipt, physical inventory, and goods issue [6]. Part of the process of opening a new store includes the choice of location(*site selection*), which is done by management, where ML algorithms can provide support. For example, Wang et al. [90] made a quantitative site selection, so that a very large number of possible store locations are reduced to a small number, which is then investigated further by qualified employees. Machado et al. [53] followed a different approach, in which a pre-selection had already been made and the remaining five stores were evaluated by a neural network. The site selection was grouped into the shell for master data with the goal of creating a master data record for the store. Another formulation of the site selection problem is when a mall is designed with a given set of sets. For example, Miao [59] used ML algorithms to find a reasonable layout for shopping malls where the relationships of stores to each other were taken into account.

### Economic-Operative Tasks

The following subsections address the economic-operative tasks of the shell model, which are the primary activities of retailers. These tasks fulfill the bridging functions and resemble a retailer’s value proposition [48]. We have identified ML application areas for the tasks of managing goods, serving customers, handing out goods, and ordering goods.

#### Managing Goods

A central aspect of the economic-operative tasks is the managing of goods, which is reflected by the large number of papers (59) on this area. The task of managing goods consists of the assortment policy, the conditions policy (purchasing price and selling price), the placement policy, and the promotion policy. In addition, managing goods also includes inventory management, which refers to the management and optimization of goods and storage respective to the inventory. Prices are an important driver that influence how often an article is sold and the resulting profit margin. This also influences inventory, since a higher price leads to lower sales, and vice versa, leading to either empty or full stock, due to varying demand or supply. Therefore retailers can implement *price optimization* so that the demand fits the supply. For example, Chandrashekhara et al. [11] determined the best prices of smartphones based on different features of the smartphones.

Furthermore, *promotion optimization* can be used to specifically increase sales for a short period of time. The general promotional effect was forecast by Henzel et al. [30]. Retailers implement *product recommendation* systems to enhance the matching of supply- and demand-side participants, which we assigned to promotional tasks and thus the task of managing goods. These systems recommend supply-side products to customers based on previous purchases, ratings, or search behavior [37]. In contrast to the traditional recommendation that suggests popular products to customers, the algorithm of these researchers aims to match customers with appropriate niche products. Thus, online shops can more specifically target the long tail [57] and exploit niche markets to generate additional revenue. To determine the ranking of products that should be recommended to demand-side participants, Liang et al. [51] performed sentiment analyses based on online product reviews from Taobao.com. As personalized product recommendations may be biased by supply-side marketing endeavors, Wan et al. [89] proposed ML algorithms to allow for product recommendations in underrepresented market segments. The authors used two data sets from online shops selling apparel and electronics. Promotional efforts can be performed for a specific group of customers. To determine such a group of customers, ML algorithms are used, which we also categorized to the promotional task. By analyzing the expert interviews, we were able to confirm the use of ML for product recommendation. Three additional processes were identified that can be supported by the use of ML. In e-commerce, ML is used to predict which items a customer will put in the shopping cart. This results in customer-specific recommendations (Interviewee 5). To increase overall revenue, ML is used to initiate cross-selling. For this purpose, a supplementary suggestion is made to the current shopping cart, based on historical data (Interviewee 4). ML is also used in both, brick-and-mortar retail and e-commerce where products are selected for promotions. This is achieved by a sentiment analysis, in which social media data are evaluated to select promotional articles (Interviewee 5).

An important application area of ML in retailing is *customer churner classification* [49]. “Customer churn” describes the loss of customers [39] who generated sales in the past and will no longer generate sales, mostly because of an active decision to generate sales with a competitor. Clustering churners categorizes demand-side participants into two clusters: one with churning customers and the other one with non-churning customers, which results in a binary classification problem [39]. While possible churners can be addressed with special marketing campaigns, the quality of services can be targeted at non-churning customers. Keeping existing customers in the sales funnel is considered less expensive than acquiring new customers [64, 79]. Customer satisfaction has a positive influence on customer repurchase behavior [36]. To implement a *customer repurchase* analysis, Kumar et al. [43] used a combination of ML techniques including both customer characteristics and e-commerce attributes. This application area allows retailers to predict whether a demand-side participant will rejoin the sales funnel, resulting in additional sales transactions and revenue.

Using an ML algorithm on e-commerce data Singh et al. [79] suggested detecting very loyal customers and providing them with higher-quality services to increase their satisfaction and to retain them in the sales funnel. Jheng et al. [36] mined the transaction logs of an enterprise resource planning system at an online shop to predict customer repurchase behavior. Moreover, Mahaboob et al. [54] develop an ML model to describe the moderating role of customer loyalty on customer retention. Predicting the probability of a lead to convert into a customer and the profitability of this new customer is crucial in retail [17]. The *customer conversion* analysis implemented by D’Haen et al. [17] applied ML algorithms to the sales data of a German business-to-business (B2B) e-commerce company and complementary data from the internet to predict the profitability of a lead. Knowing which leads will be profitable can help retailers to directly address these leads and increase their conversion probability. Niu et al. [65] applied multiple ML algorithms to predict the probability of a customer purchasing a product based on his or her (previous) search behavior. The computational model was applied to e-commerce data from Walmart. Thus, a retailer can predict a customer’s willingness to purchase and pay in an early stage of a sales funnel [9].

#### Serving Customers

The following application areas are concerned with tasks that serve customers. In brick-and-mortar stores, the products are made available using limited shelf space. The digital equivalent is online stores, which often provide a much larger assortment. Therefore, a product *search function* function is necessary to serve customers based on their demand, which is the reason we categorized the product search function as a business task. Khatwani et al. [38] developed a model for predicting customers’ individual information search preferences using questionnaire data.

To enable a good product search, products must be classified first. E-commerce *product classification* challenging, due to the large scale and complexity of product information and categories. Yu et al. [96] combined multiple ML algorithms to propose an e-commerce text classification. As reviews are the online counterpart to the recommendations of an employee, we categorized review analysis with ML algorithms to the economic-operative task of serving customers. To increase a retailer’s role as a trustee, retailers often implement systems to review the transaction partner and the products sold. Suppliers and customers of a retailer are aware of reviews, as these often directly impact their business [32]. Vinodhini and Chandrasekaran [87] implemented *review classification* based on customers’ opinions (opinion mining). They used publicly available customer reviews and classified these into positive and negative reviews, so that supply- and demand-side participants can preselect reviews possibly more relevant for them.

Opinion spammers and fake reviews exploit customer trust and harm the reputation of e-commerce retailers as a trustee by posting false or deceptive reviews [97]. These reviews are difficult to detect because of complex interactions between several user characteristics [45]. To enable *fake review detection* Hussain et al. [32] implemented a behavioral method that utilizes 13 different behavioral features to calculate a review spam score for each reviewer. As review rankings are an important indicator of the relevance of a reviews to demand-side participants, the *review consistency* between the review ranking and the review summary is crucial. To verify this consistency, Zhang et al. [97] proposed an ML approach based on e-commerce data. This approach enables demand-side participants to identify relevant reviews based on the review rankings. As reviews are often created by other customers, shopping assistants are more responsive to the needs of a special customer and therefore the interaction with an employee is digitally and automatically mapped.

Bertacchini et al. [7] developed a *robotic shopping assistant*. These robotic assistants can promote sales in general and guide sales decisions. A shopping assistant can also be implemented on a customer’s smartphone as a companion app [94]. Many online shops offer a 24-hour service using *lingual shopping assistants*. This service is very expensive when done manually. Chatbots can be used as a solution for automatic online shopping, but the chatbot has to be able to give accurate and quick answers. For example, Nursetyo et al. [66] proposed an intelligent chatbot system that can be used as an e-commerce assistant and supports the process of serving customers.

#### Handing out Goods

The business task of handing out goods includes the transaction in which the product becomes the customer’s property. This process can be improved or automated with ML algorithms. Kourouthanassis and Roussos [40] developed an *unmanned and automated checkout system* with a smart shopping cart. Shopping carts are unusual in the fashion industry segment, but, for example, Hauser et al. [28] developed an ML algorithm to determine whether a product had passed the store exit or was only registered because it was placed near the gate-mounted antennas. Suponenkovs et al. [80] developed a system for *automatic invoice recognition* by analyzing the pixels with respect to their relevance and texture analysis. As an offline scenario, payment for fruit or vegetables in retail stores normally requires them to be manually identified. Rojas et al. [72] and Femling et al. [20] presented image classification methods with the goal of speeding up the checkout process in stores.

Since incorrect operations or failures by employees at points-of-sale (POS) account for 24% of inventory differences in the retail industry [18], ML algorithms are used to reduce these differences. *Fraud detection* is assigned to the business task of handing out goods, since problems with the transaction in which the product becomes the customer’s property should be avoided. For interim transaction fraud detection at checkouts, Trinh et al. [82] developed a method that uses image recognition to determine the hand movements of cashiers. It should be recognized by the algorithm whether all articles that are handed out to the customer are also recorded in a sales process, that is, on the receipt. Fraud detection in retrospect must be dealt with in a human resources management process. Pehlivanli et al. [69] pursued a different approach by analyzing transaction data. ML algorithms were trained to classify fraud and non-fraud on the basis of indicators such as profitability, stock turnover, stock cost, and shelf life. This process was supported by our findings from the interview analysis. According to the interviewees, ML can be used to detect anomalies in stock transfers by evaluating order times and quantities, as well as a store’s inventory situation, and then making a prediction regarding missing delivery quantities (Interviewee 4). Moreover, ML is implemented to perform an evaluation regarding the reservation of products and their actual collection. This can minimize the risk of blocking inventory (Interviewee 6). Furthermore, ML is used to classify the credit scores of customers in e-commerce. Kulkarni and Dhage [42] used ML and data mining techniques to integrate information obtained from social media to protect retailers from payment defaults. In addition, the interviewees provided insight into the audit process, where ML is used to support retailers in fraud detection. Here, ML is used to check invoices for consistency and correctness and to detect any anomalies in invoices (Interviewee 1).

#### Ordering Goods

We identified out-of-stock management and supplier analysis as application areas of ML for the ordering of goods tasks. “Out-of-stock” describes shelf space in brick-and-mortar stores that is no longer filled by the intended article and is empty. Customer demands can no longer be fulfilled. As a *detected out-of-stock* triggers the replenishment process, this application area is assigned to the business task of making goods available. For example, Paolanti et al. [68] implemented a mobile robot using visual and textual analysis to facilitate the automatic detection of shelf out-of-stock situations. The use of ML in out-of-stock management can also be seen in three processes that we were able to extract from the expert interviews. For example, ML can be used to perform zero-stock forecasting, so that goods can be moved and redistributed between various stores depending on actual inventory levels. In distribution, ML can be used to predict and plan inventories. A prognosis of delivery delays is created and then used to predict when an item will be out of stock (Interviewee 6). Another Interviewee explained the usage of ML in out-of-stock management where a forecast of spoilage of goods takes place. For this purpose, historical data are used to minimize deprecation (Interviewee 8). A problem for the retailer is when item inventories are present for more than one day with zero inventory according to the system until the next delivery. These items are out-of-stock items in the store and can be predicted using ML by evaluating the order time and order quantity, to create a risk representation for specific items (Interviewee 1).

Once the replenishment process is triggered, a supplier must be selected. Kuo et al. [46] considered the aspect of the sustainability of suppliers in their *supplier selection* optimization. ML algorithms are used to resolve supplier selection problems regarding operational performance and environmental issues in terms of the criteria of corporate social responsibility, service, cost, environment, quality, and delivery. This was also confirmed by our interview analysis, in which we identified a process of ML use in the course of inventory management. For inventory management, ML can be used to analyze historical point-of-sale data and past inventories to identify anomalies in the movement of goods (Interviewee 4). In inventory planning, ML can be used to forecast potential delivery delays, using historical deliveries for delivery time and amount of goods. This helps in the planning and procurement of goods (Interviewee 3). Another possible application of ML in the context of retailer/supplier analysis is the evaluation of parcel service providers with regard to shipping day, delivery day, and volume, in order to be able to create a forecast for delivery reliability (Interviewee 6). In addition, ML can be used to make a statement about supplier delivery reliability by evaluating the delivery day according to the order and the actual delivery day (Interviewee 4).

In addition to the results of the structured literature research, we were able to identify a further application area for the use of ML in the context of economic-operative tasks, one that is primarily concerned with the procurement and replenishment of goods. Procurement and replenishment is strongly interlinked with the other application areas but does not uniquely belong to one area, thus we chose to see it as a separate application area. It mainly focuses on the timed ordering of goods which can be combined with out-of-stock management to show only one interlinking. Thus, we identified a total of seven different processes which can be supported by the use of ML. The task of procuring goods deals with the timely reordering of articles in order to avoid a situation of empty shelves. On the other hand, however, only as much should be reordered as can actually be sold, so that there is no overfilling of the shelves, leading to associated higher storage costs. For this purpose, current inventories as well as historical goods movements must be taken into account. In the fashion sector, ML can be used to create seasonal forecasts and thus minimize depreciation. Some of the items will then be carried over to the next season and possibly priced differently (Interviewee 3). Other possible uses of ML in this area are automated stock transfers, for which a forecast of future zero stock must first be created, after which the articles can be distributed evenly across all stores. Another use of ML is to determine a sales forecast per price, first in order to avoid zero stock at a low price or slow-moving items at a high price (Interviewee 7), and second, to create an action forecast based on point-of-sale data to ultimately perform targeted merchandise management (Interviewee 4). In order to examine stocks in more detail, the use of ML can also help. Anomalies within the inventory can be identified in order that the retailer can be informed in which product areas action is required (Interviewee 3). Another option is to analyze the capacity of a company’s employees. This can be particularly helpful in workforce planning to determine how many employees can be deployed within a store or warehouse (Interviewee 7). In procurement, ML can also be used to optimize resources. This concerns both own and external capacities. Above all, in production the resources can be optimized in such a way that machines are ideally used up, by, for example, products being pre-produced (Interviewee 4). The last process we were able to extract from the analysis of the interviews concerns contingency. Here, ML can be used at several points within the process, for example, by performing buyer prioritization. This, in turn, is used to allocate inventory to appropriate customers (Interviewee 7).

## Demonstration of Machine Learning Application in Selected Retail Processes

This section presents two selected processes based on the reference process description by Becker and Schütte [6], and the use of ML is demonstrated for the corresponding activities extracted from the interviews. We selected a return process and a stock relocation process to demonstrate the application of ML in retail.

**Fig. 5:**
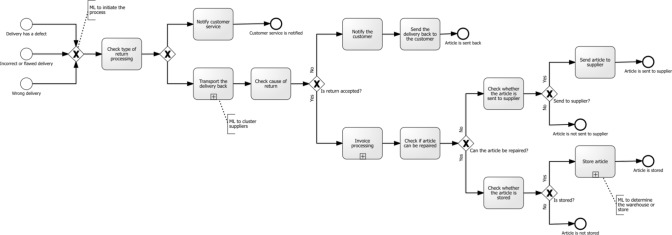
The Return Process in BPMN

The first process deals with *customer returns* as illustrated in Fig. 5. Normally, the return process begins with a call from the recipient to notify distribution about an incorrect, defective, or wrong delivery. At this point, ML can be used to calculate the likelihood of the recipient initiating this call, based on the customer’s historical returns (Interviewee 3). Next, distribution decides how the return will be managed. If it is not possible to fix the problem with the help of customer service, the article of the delivery have to be transported back. Based on an analysis of different suppliers, ML can create a score to cluster suppliers by returns and thus integrate them into the return process (Interviewee 6). The recipient can bring the article back themselves, or the article can be picked up. If the return is then declined, the delivery is sent back to the customer, who gets a notification. In the case of accepting the return, the invoicing process is started, and it is checked whether the article can be repaired or not. If the article cannot be repaired, the article will be sent back to the supplier, or it will be disposed of. If the article can be repaired, it will be fixed and stored in a warehouse. At this point, ML can be used to determine the warehouse or store where it will be sent by creating a zero-stock forecast (Interviewee 2).

**Fig. 6:**
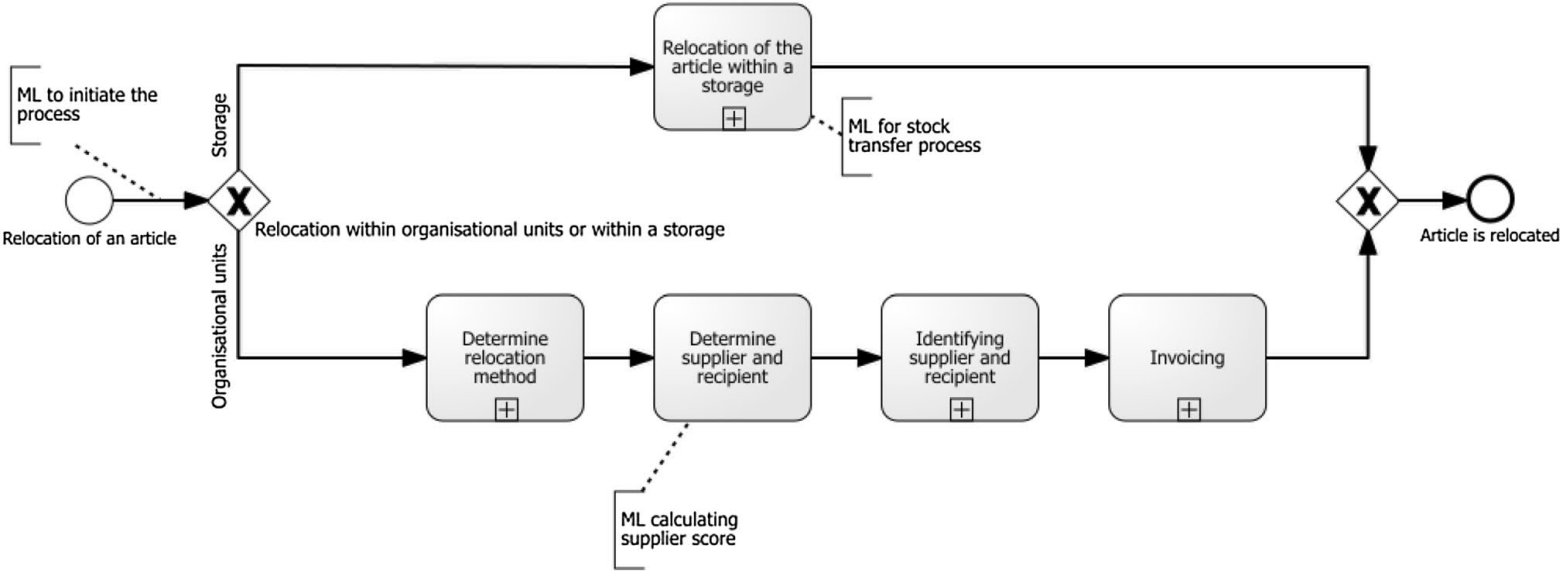
The Stock Relocation Process in BPMN

The second process deals with the *stock transfer of goods* as illustrated in Fig. 6. This process can be initiated after analyzing the current stock and calculating the probability that an article will be out of stock (Interviewee 2). This means an article has to be relocated to a store where it may be out of stock in the future. If an article has to be relocated, then it has to be decided whether it is to be relocated to a storage or organizational unit. In the latter case, the relocation method has to be chosen, and often a relocation with invoicing is chosen. The stock transfer process can be automated by creating a zero-stock forecast (Interviewee 2). Next, the supplier and recipient have to be determined, which then leads to several processes involving the identification of these. The supplier could be a storage unit, customer, or store, while the recipient could also be a storage unit, customer, or store. This involves the use of ML when choosing a customer as supplier. The customer can be given a score based on an evaluation of his or her historical data and, based on this score, the supplier is then determined (Interviewee 4). After this, the invoicing is done. Lastly, the article is relocated, both in the case of storage and organizational units; this is the end of the process.

## Discussion

### Summary of Key Contributions

Our structured literature review, as well as the analysis of the expert interviews and the subsequent structuring of the ML application areas along the retail-related tasks depicted in the shell model (Fig. 1), led to the development of a framework for determining possible ML applications in retail (Fig. 4). Our structured literature review and interview analysis on ML application areas in retail resulted in three major findings.

First, a closer analysis of the framework reveals that there is a trend to use ML to support decision-oriented and economic-operative tasks in retail [77]. Other crucial tasks in retail, such as master data management as well as technical and administrative tasks, are either not covered by ML applications or underrepresented in our literature sample. On the one hand, the support and automation of economically valuable tasks seem reasonable, as they represent the main bridging functions and the major value proposition, can be used to differentiate a company from its competitors, and are a major source of revenue for retailers [77]. On the other hand, master data and technological facilities consist of the necessary data required for ML algorithms [92]. If these data are inconsistent or not even available, ML models provide false results or can even not be trained.

Second, our integrative approach for the review in brick-and-mortar retail and e-commerce is relatively balanced regarding the application environment, with 107 papers focusing on offline retail and 118 papers focusing on online retail. The papers also reveal that ML applications make use of the peculiarities of each environment. While the selection of an optimal site for a brick-and-mortar store or the support of unmanned checkout systems with ML concern offline retail, ML-based analyses of customers and product reviews are only possible in e-commerce scenarios in which these data exist [32]. As mentioned earlier, e-commerce involves much data regarding its customers and unstructured data regarding products. This process will possibly be driven in future by VR (virtual reality) shopping. Therefore, in addition to click and mouse movements in e-commerce, also hand, eye, head, and leg movements could be tracked [95]. We assume that the existing application areas presented in this work, especially from e-commerce, can be adapted to VR shopping, and future research may find new application areas, especially for VR shopping [95].

**Fig. 7:**
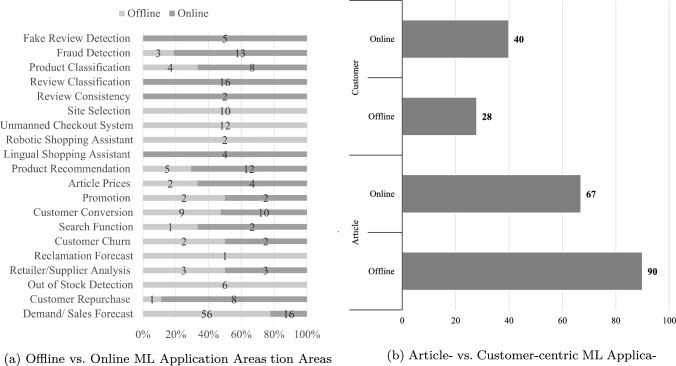
Application area analysis based on literature analysis [34]

However, we also identified application areas that can be applied to ML in both environments, such as customer conversion, promotion optimization, and product classification (Fig. 7a). This trend is also supported by the fact that brick-and-mortar retailers are increasingly integrating technology within their stores to bring in the online convenience and experience [16, 74, 94]. Product placement is a task necessary to optimize revenues in brick-and-mortar stores, but it can also be used in electronic shops to optimize the placement of a product on a website [33]. As these ML models can be used in both environments, the effort of implementation can be balanced by the dual application. Thus, these applications are also useful for retail using a multi- or omnichannel approach [84].

Third, the analysis of the application areas for the object of interest reveals a tendency towards the customer-centered application of ML in e-commerce (40 papers), while the focus in the offline environment is on a single or set of articles (90 papers) (Fig. 7b). Our analysis also confirms the developments described by Grewal et al. [26], towards a more customer-centered focus in brick-and-mortar retail (28 papers). However, we propose to focus even more on the customer for proper ML implementation and address the customer experience for value generation [24, 85]. To select an optimal site, it is necessary to identify a location with an adequate number of potential customers and to select the right articles for the needs of a specific customer milieu [90].

### Limitations and Outlook

This research has certain limitations, which at the same time open interesting avenues for future research. As previously mentioned, the proposed framework for ML application areas in retail does not cover master data management or technical and administrative tasks. These should be investigated by future research. We concentrated on retail as the domain of application for ML. Retail can be distinguished from wholesale by small units, trading net prices, and operating using a business-to-customer business model [48]. Although our framework for ML application was developed specifically for retail, we propose that many of the ML applications can also be used in a wholesale context, as the tasks are broadly similar (i.e., bridging functions) when comparing retail and wholesale architectures [77]. We intentionally focused our literature research on ML applications in scientific publications to ensure the maximum level of quality and comprehensibility of our research. However, we are aware that implementations in practice can be ahead of scientific publications [19]. Hence, we conducted interviews with eight domain experts, knowledgeable about retail and enterprise systems. In addition, we aim to integrate practitioner sources, such as company white papers and publications by retail interest groups, into future research. In preparation for the expert interviews, we used the preliminary application areas resulting from our literature review and the tasks summarized in the shell model [77]. This might have led to a potential bias in the selection of the interviewees, although we had the impression that the interviewees reported their experience of the application of ML in retail depending on their domain of interest. For the literature review, we opted for a broad research scope, reflected in our search string and the databases queried. However, we did not provide a more detailed analysis of the algorithms and data used in each ML application. An important avenue for future research would be to detail this broad overview and to provide evidence of proper algorithms to be implemented within each application area. As we focused on identifying ML applications in retail, we used a task-oriented architecture developed by Schütte [76] that focuses on the business layer as a starting point. However, we are aware that there are other reference architectures covering additional application and technical architecture layers in general and integrating ML in particular [3]. Although these architectures include additional layers, they are less detailed with regard to retail-specific tasks. It might, therefore, be worthwhile for future research to extend the existing task-oriented architectures [6, 77] with technical layers integrating ML. Alternatively, an architecture may profit from providing interfaces to plug in additional services (e.g., ML services), making the architecture more flexible [2].

## Conclusion

We have identified 21 application areas of ML in offline and online retail, based on a thorough analysis of the current body of literature and analyzing eight expert interviews. The application areas identified cover decision-oriented (business intelligence) and economic-operative tasks (managing goods, serving customers, handing out goods, ordering goods). Based on our analysis, ML can be implemented to support and automatize structured and unstructured tasks in retail. In addition, current research is equally concerned with the application of ML in offline and online retail. For e-commerce, we identified a tendency for the customer-centric use of ML, while in the brick-and-mortar context, the retail article is more often the object of interest. The contribution of our paper for practitioners and researchers is a general overview of current research on ML applications in retail. For practitioners, the framework for ML application can be used by retailers to determine which tasks ML can support in a company. Thus, the framework can be used as an indicator for the usefulness of an ML application and to determine whether an ML implementation project is feasible and makes sense from an economic perspective. The algorithms developed and applied in the identified papers can also serve as a starting point for the practical consideration of implementation. In addition, the interview analysis provided insights into where ML is actually used in retail and can be applied to increase the revenue and efficiency of companies, as well as to improve the processes involved in economic-operative and decision-oriented tasks. For researchers, this work provides a retail-specific framework for ML application to foster the development of more holistic and integrated ML models (e.g., promotion-sensitive resource optimization).

## Data Availability

The data that support the findings of this research article contain corporate information and restrictions apply to the availability of these data. Anonymized summaries of the interview data are, however, available from the authors upon request.
